# Changes in cannabis policy and prevalence of recreational cannabis use among adolescents and young adults in Europe—An interrupted time-series analysis

**DOI:** 10.1371/journal.pone.0261885

**Published:** 2022-01-12

**Authors:** Alexander Carl Gabri, Maria Rosaria Galanti, Nicola Orsini, Cecilia Magnusson

**Affiliations:** 1 Department of Global Public Health, Karolinska Institutet, Stockholm, Sweden; 2 Centre for Epidemiology and Community Medicine, Region Stockholm, Stockholm, Sweden; University of the West of Scotland, UNITED KINGDOM

## Abstract

**Background:**

Cannabis policy varies greatly across European countries, but evidence of how such policy impacts on recreational cannabis use among young people is conflicting. This study aimed to clarify this association by investigating how changes in cannabis legislation influenced cannabis use.

**Methods:**

Available data on self-reports of recreational cannabis use among individuals aged 15–34 years was retrieved from EMCDDA. Information on cannabis policy changes was categorized as more lenient (decriminalisation or depenalisation) or stricter (criminalisation, penalisation). Countries that had implemented changes in cannabis legislation or had information on prevalence of use for at least eight calendar years, were eligible for inclusion. We used interrupted time-series linear models to investigate changes in country-specific trajectories of prevalence over calendar time and in relation to policy changes.

**Results:**

Data from Belgium, Czech Republic, Germany, Italy, Netherlands, Norway, Portugal, Slovakia, Spain, Sweden and United Kingdom, for 1994–2017 was available for analyses. Cannabis use varied considerably over the study period and between countries. On average, use was stable or weakly increasing in countries where legislation was not changed or changed at the extremes of the study period (+0.08 percent per year [95% CI -0.01, 0.17 percent]). In contrast, the pooled average use decreased after changes in legislation, regardless of whether it had become more lenient (-0.22 [-1.21, 0.77]) or stricter (-0.44 [-0.91, 0.03]).

**Conclusions:**

Our findings do not support any considerable impact of cannabis legislation on the prevalence of recreational cannabis use among youth and young adults in Europe.

## Introduction

Cannabis is the most commonly illicit drug used worldwide with an estimated 192 million users in 2018 [1]. In Europe, about 90 million individuals aged 15–64 years used cannabis once or more during their lifetime and almost 1 in 10 young adults were monthly users during 2019 [2].

Cannabis use and sales may have considerable social and public health consequences. A monthly use has been associated with increased risk of psychosis, injuries, and poor obstetric outcomes when compared with non-user populations [3–6], as well as poor academic performance and decreased motivation [6–8]. Additionally, it has been recommended by the World Health Organization that cannabis is rescheduled in the international drug control framework from Schedule IV (particularly harmful and with few therapeutic properties) to Schedule I (especially serous risk to public health and limited if any therapeutic usefulness) [9]. While cannabis’ health effects remain disputed, cannabis has been found addictive and may be associated with the risk of other substance use disorders according to a study drawing from national surveys by the US “National Institute on Alcoholism and Alcohol Abuse” [10].

Considering these possible pervasive effects of recreational cannabis use on society, the dearth of any international treaty harmonising its regulation is problematic [11, 12]. Although there is an obligation for nations to control the cannabis plant, the framework of the United Nations 1961 single convention on narcotic drugs is considered ambiguous, due to the specific exclusion of “possession of cannabis for personal use” from EU legislation, resulting in each individual member state hosting administrative responsibilities for such offences [13]. In line with this ambiguity, the international Narcotics Control Board is championing a strictly prohibitive interpretation of the UN convention while there is a wave of policy liberalisation throughout the world [13].

No country in Europe has to date legalized (i.e permitted personal use and supply) cannabis [12], in contrast to several jurisdictions in the Americas and Australia [14–16]. Instead, several European countries have relaxed policies by either decriminalisation or de-penalising use and possession. Nevertheless, cannabis policy still varies considerably across Europe. While Sweden enforces criminal prohibition and has adopted a vision of “a drug free society”, Belgium, Portugal, Estonia and more recently the Czech Republic have decriminalised use. The Netherlands and Spain have semi-legalized approaches, with sales and use being accepted by some regions within the Coffeeshop- and Cannabis Club systems, respectively. The variation is likely to reflect both the lack of consensus regarding which line of cannabis policy holds the best outcome concerning public health, economy, and criminal activity [17, 18] and variations in cultural attitudes to cannabis.

The “rational choice theory” [19], is one existing conceptual framework first formulated by Clarke & Cornish in 1986, which stipulates that people are rational with a self-interest driven mind set capable of employing risk-reward estimations. Based on this theory, legislators have historically led punitive drug campaigns in the belief that cannabis use may be greatly influenced by fear of prosecution, because individuals may integrate societal norms (e.g. punishment, harms to others) in their risk appraisal [19]. It may, however, also be true that market-induced levels of recreational cannabis use influence societal norms that in turn shape drug policy [20, 21]. Lastly, social and economic processes in micro- and macro- environments may affect cannabis use independently of legislation [22–24], including market variables influencing the price and availability of cannabis [25].

Previous research on the impact of cannabis legislation on prevalence of cannabis use among adolescents and young adults in Europe [23, 26], but also elsewhere [19, 27–30] is inconclusive. This knowledge gap may be explained by methodological obstacles, including difficulties in obtaining representative, population-based, samples and differential underreporting of drug use in surveys because of stigma [30, 31].

To overcome this knowledge gap, we here take advantage of unique, and already collated cannabis prevalence data from the EMCDDA to explore the impact of changes in national cannabis policy on levels of use in adolescents and young adults in Europe. We apply an interrupted time-series approach to disclose the influence policy change more fully per se, to inform the current debate on this matter.

## Methods

We retrieved data on prevalence of cannabis use among young adults and adolescents in European countries, as gathered and harmonized from various national surveys by the EMCDDA [32]. We included countries where data for at least eight calendar years was available, or where changes in cannabis policy had been implemented. To avoid including sporadic use in the prevalence measures we focused on “past month use” that is more likely than the broader “past 12-month use” to reflect current (and recurrent) behaviour. Thus, we included self-reported information on “cannabis use in the past month” among individuals aged 15–34 years from Belgium, Czech Republic, Germany, Italy, Netherlands, Norway, Portugal, Slovakia, Spain, Sweden, and United Kingdom use in our analyses.

For quality assurance, we cross-checked prevalence estimates between the EMCDDA data repository and available reports (see S1 File), and definitions of study populations and response rates over time and between countries. Details regarding methods for the original data collections in the various countries are available from the EMCDDA data repository web site [32].

We identified national narcotic policies from a recent EMCDDA report [2], and from governments’ official web sites. We categorised changes in national cannabis policies as “more lenient” when decriminalisation (i.e. reinstatement/removal of criminal status from a certain behaviour or action, which does not denote said act as legal as non-criminal punishments may still be applied) or depenalisation (i.e. introduction of the possibility of closing a criminal case without imposing punishment) of cannabis use, possession or acquisition for personal use had been implemented. Conversely, we categorized such changes as “stricter” when criminalisation and/or penalisation had been implemented. These definitions were based on suggestions from the EMCDDA [2]. Details of the specific policy changes are listed in S2 File.

### Statistical analysis

We used interrupted time-series linear models to investigate changes in trajectories of self-reported prevalence of cannabis use over calendar time overall and in relation to policy changes [33]. The amount of data varied considerably across countries. Country-specific prevalence estimates were available for between 4 and 19 calendar years, policy changes within each country ranged from 0 to 2, and the calendar year of such changes, if any, varied across countries.

For the three countries (Czechia, Italy, and UK) where policy changes had been implemented and data points were available before and after intervention, calendar time was centred around the year of policy change within each country. Years from policy change was then modelled with a linear function before intervention and a linear spline with a knot at 0 year (legislation change). The regression coefficient of the linear spline represents the change in the linear trend of self-reported cannabis use after legislation change. To take into account heterogeneity across countries, random-effects were introduced in the constant, the regression coefficient of the linear trend before intervention, and the regression coefficient of the linear spline function.

In the remaining eight countries without legislation changes (Germany, Netherlands, Slovakia and Sweden) or where the change had been implemented at the extremes of the period with data (Belgium, Norway, Portugal, Spain), calendar time was modelled with a linear function with random-effects in both the intercept and the regression coefficient of the linear term.

Country-specific linear trends were derived from the estimated mixed-effects model using the best linear unbiased predictions (BLUPs) of the random effects. Wald-type test of hypothesis and confidence intervals for the fixed-effects, representing average trends across countries, were obtained from mixed-effects model fitted via restricted maximum likelihood method.

## Results

**Fig 1:**
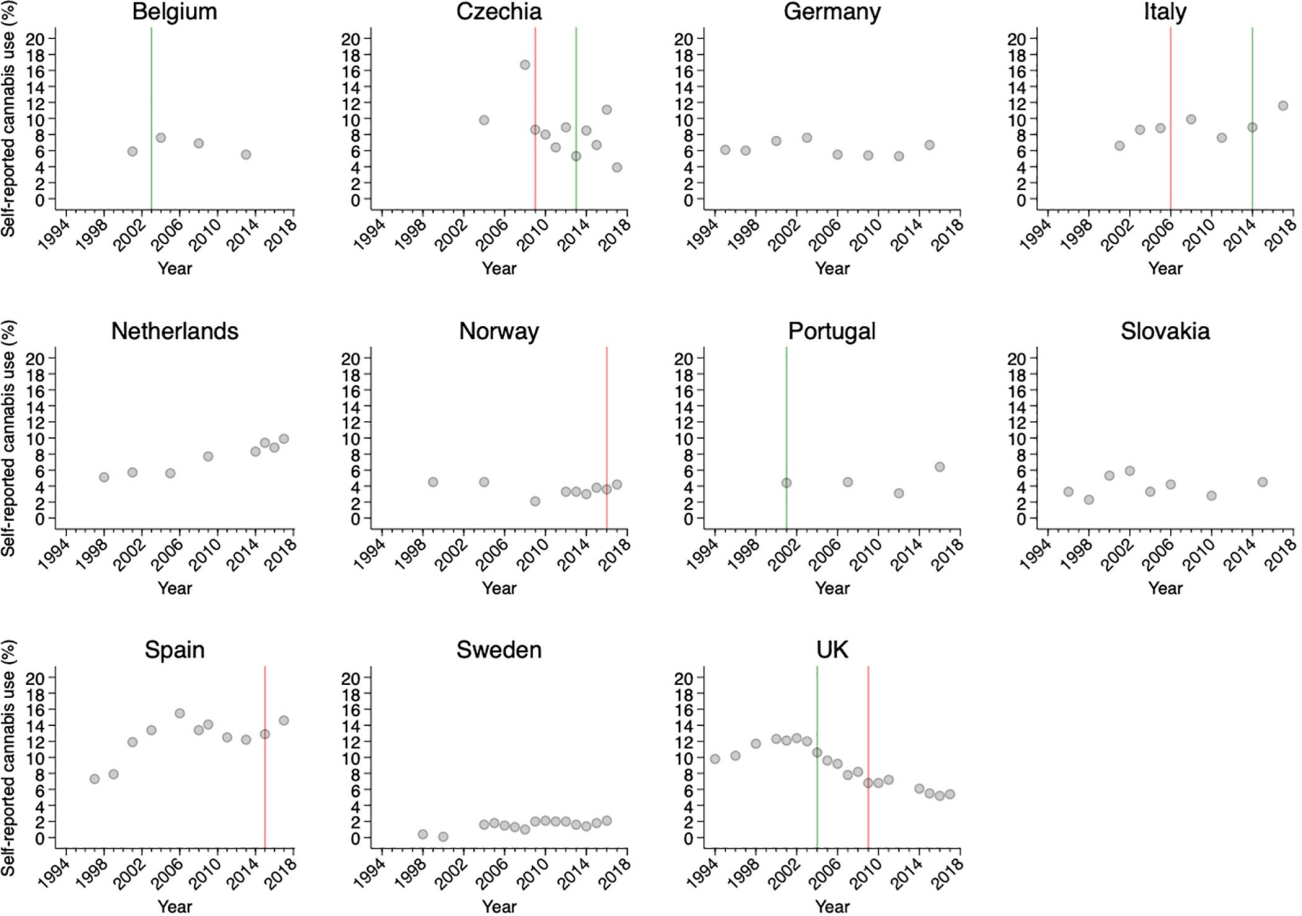
Country-specific prevalence of self-reported cannabis use in the last month among individuals aged 15–34 years in Europe, by country [32]. The implementation of more lenient and stricter legislations is indicated with green and red lines, respectively.

The available country-specific data on self-reported cannabis use during the last month among young adults and adolescents in 1994 through 2017 are presented in Fig 1. The prevalence of use varied greatly over time and between countries. Spain (12.3%) and Sweden (1.5%) had the highest and lowest averages of self-reported use during the study period. Changes in cannabis legislation are also illustrated in Fig 1 and presented in detail as S2 File. In Czechia and Italy, a stricter legislation was followed by a more lenient legislation (red vertical lines are preceding the green lines). In UK, it was the opposite.

Cannabis use was either stable or increasing among countries where cannabis legislation remained unchanged (Germany, Netherlands, Slovakia, Sweden) or had been implemented at the extremes (Belgium, Norway, Portugal, Spain) of the study period. On average, self-reported cannabis use seemingly increased by 0.08 percent per year (95% CI = -0.01, 0.17 percent) in these countries.

**Fig 2:**
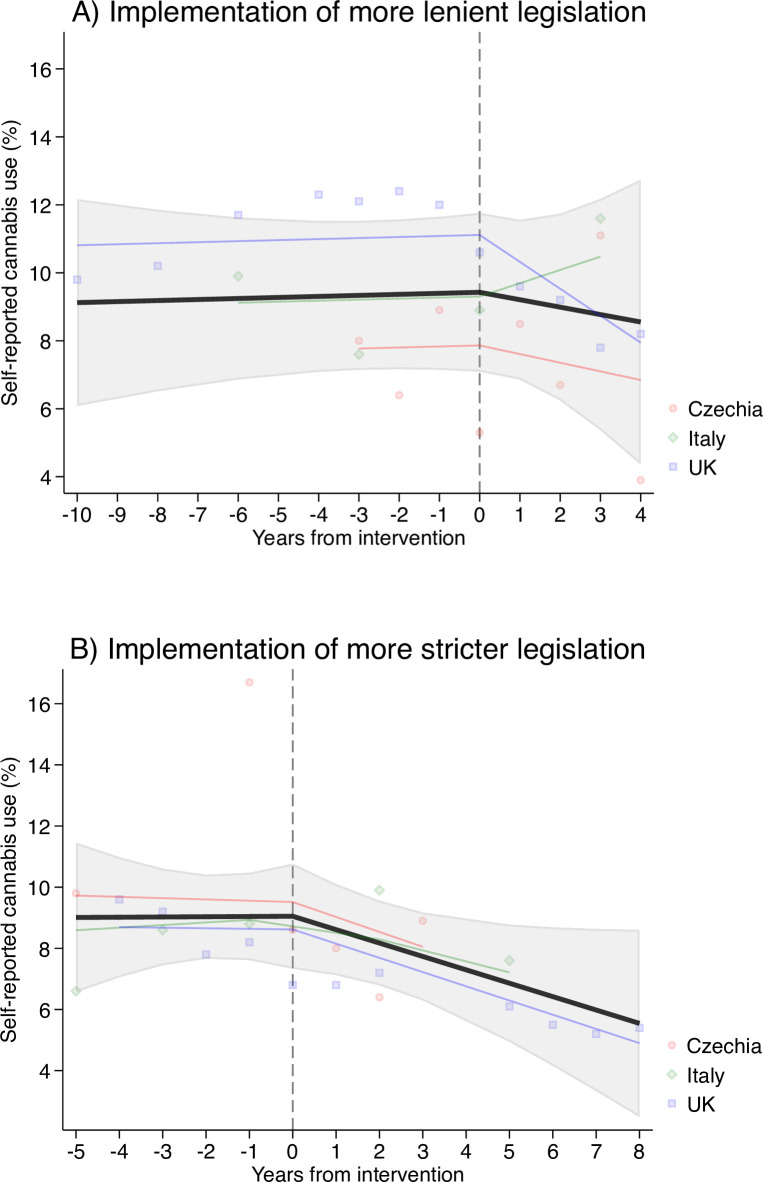
Self-reported cannabis use (%) and linear trend estimates (black line is the average across Czechia, Italy, and UK) and country-specific predictions (coloured lines) before and after more lenient (Panel A) and stringent (Panel B) legislations. Years from intervention was modelled with a linear spline with a knot at 0.

In contrast, use of cannabis appeared on average to decrease in the three countries where cannabis legislation had changed during the observable interval of the study period, regardless of whether it had become more lenient (panel A, Table 1 and Fig 2) or stricter (panel B, Table 1 and Fig 2).

**Table 1:** Linear trends and confidence intervals estimated with mixed-effects models for self-reported prevalence of cannabis use, before and after changes in cannabis legislation in the Czech Republic Italy, and UK.

Country	Trend before legislation change, % per year (95% CI)	Trend after legislation change, % per year (95% CI)	Year of legislation change (period interval)^c^
*A) Implementation of more lenient legislation*			
All countries^a^	0.03 (-0.26, 0.32)	-0.22 (-1.21, 0.77)	
Country-specific^b^			
Czechia	0.03	-0.25	2013 (2010–2017)
Italy	0.03	0.39	2014 (2007–2017)
UK	0.03	-0.79	2004 (1998–2008)
*B) Implementation of stricter legislation*			
All countries^a^	0.01 (-0.61, 0.62)	-0.44 (-0.91, 0.03)	
Country-specific^b^			
Czechia	-0.04	-0.49	2009 (2004–2012)
Italy	0.08	-0.36	2006 (2001–2013)
UK	-0.02	-0.46	2009 (2005–2017)

^a^ The estimates of the linear trends (fixed-effects are average across the 3 countries) and 95% confidence intervals were obtained with a mixed-effects model with calendar time centred about the intervention year. A linear spline with a knot at 0 was used to detect the possible linear change after intervention. Random-effects were introduced in the intercept, linear trend, and linear spline to take into account possible heterogeneity in outcome trajectories across countries.

^b^ Country-specific linear trends (Best Linear Unbiased Predictions) before and after intervention were computed from the estimated mixed-effects model obtained using restricted maximum likelihood method.

^c^ Index of legislation changes for each country may be found in S2 File.

On average, self-reported cannabis use decreased by 0.22 percent (95% CI = -1.21, 0.77) per year after legislation had become more lenient in these countries. This estimated average decline accorded well with the hypothesis of no legislation effect (z = 0.43, *p*-value = 0.667). Country specific trends were heterogenous as use appeared to increase in Italy while it declined in Czechia and the UK after policy change (Fig 2 Panel A).

Similarly, the average prevalence of use decreased by 0.44 percent (95% CI = -0.91, 0.03) per year after a stricter legislation change in the same countries. This decline was steeper than after implementation of more lenient policies, and present in Czechia, Italy, as well as the UK. Nevertheless, estimated average decline was compatible with the hypothesis of no legislation effect (z = -1.83, *p*-value = 0.067).

## Discussion

This comprehensive re-analysis of all available data from EMCDDA does not corroborate an impact of changes in cannabis legislation on cannabis use among young people in Europe. Overall, since the 1990’s, self-reported use appeared to increase among countries without any policy changes but decrease after both decriminalisation and depenalisation of cannabis-related crimes.

Our findings are in line with previous research not demonstrating any clear relationship between cannabis policy and its use, for instance a recent meta-analyses by Kotlaja et al. which measured “past month use of hashish” among 12 to 15 year olds in 27 countries; and Melchior et al. which observed no association between cannabis policy and recreational use by people under 25 years of age in data pooled from 41 articles [19, 30]. They do, however, contrast with other reports such as Shi et al., showing that depenalisation of cannabis-related crimes may increase its use on a yearly and monthly basis among 15 year-olds in northern America [34].

Following the Rational Choice Theory, the anticipated outcome of a harsher drug policy is a decrease in the prevalence of use. Data from three countries in this study, seemingly support this expectation. However, we also found a decrease in use after change to a more lenient policy. There are several explanations for a potential discrepancy between anticipations based on theoretical assumptions and the observed population trends. First, behavioural changes may follow threats of punishment to a larger extent than they do follow an increase in permissiveness, above all because these cues are likely to impact differently on different segments of users. It is conceivable that occasional and recreational users (the majority of users and of responders to surveys) remain relatively insensitive to permissiveness but value the risk of penalty rather high and not worth its reward [35, 36]. The opposite could be true among frequent users (probably the majority of non-responders to surveys), among whom poly-drug use and dependence is high and the contiguity to illegal market more established [19]. Second, the Rational Choice Theory may best apply to older adults, who have a fully developed judgement capability and impulse control. Several lines of research have suggested the immaturity of the frontal cortex typical of adolescence and young adulthood as the psychobiological cue to impulsivity and risk taking, including substance use [37]. The age group included in this analysis may be composed by a high proportion of these risk prone individuals, naturally resilient to general norms and highly sensitive to peer-group norms [23]. This would explain the overall poorly discernible cross-country patterns of use following legislation. Third, and more important the reflection of policy on observed prevalence changes may be double-edged. On the one side a harsher policy could reflect in a decreased tendency to report cannabis use in surveys, instead of or irrespective of a real decrease in use. The opposite would be true following decriminalization or depenalization: more young people would feel comfortable to report use in surveys, thus reflecting in an apparent increase in use. This possibility has received some support from studies showing a temporary increase of self-reported use during the first one-two years after policy relaxation, but not in the longer run [27, 38]. Finally, concurrent processes at the societal level (e.g. economic instability and poverty levels, immigration) may impact on substance use prevalence independently from or in interaction with legislations [24]. Whatever the explanation, it appears clear that complex population processes likely to reflect in observable trends can hardly be predicted by simply equating a change in explicit norms to a change in implicit group-specific norms [19].

Our findings should be interpreted considering some limitations. Most importantly, the duration of follow-up was insufficient for most of countries since changes in cannabis policy, if any, had occurred at the extremes of the study period or near in time. Changes in policy were also implemented in different calendar years, such that other time-varying contexts of potential importance for cannabis use could not be considered. In addition, we could solely address the gross categories of “stricter” versus “more lenient” policy changes, entailing heterogenous changes in drug penalties. Further, based on the sources we managed to trace and the EMCDDA handbook, the study quality varied between the individual surveys (S1 File) [39]. We noted that some surveys had methodological problems that either decreased transparency or affected external validity and comparability between studies. For example, there were sometimes mismatches between the age range of the survey and the EMCDDA template, response rates were sometimes low or missing, and changes in survey methodology had occurred between calendar years. We cannot exclude that these methodological shortcomings may have biased our findings.

We suggest that future studies should repeat our work when further follow-up of cannabis use after recent changes in legislation is available. We also propose that future studies on the benefits or risks of cannabis policies should evaluate a comprehensive set of indicators encompassing morbidity, health care use, and characteristics of drug markets. Lastly it would be of interest to compare trends of high and low frequency of cannabis use in response to policy changes, to understand if drug policy is likely to impact differently on these behavioural patterns.

## Conclusion

There is no evidence as of date, considering available data and previous publications, that policy changes regarding recreational cannabis significantly affect the prevalence of recreational cannabis use among young people in Europe.

## Supporting information

S1 File References describing methods for individual surveys. (DOCX)

S2 File List of implemented changes in cannabis legalisation changes during the study period, per studied country and calendar year. (DOCX)

S3 File (PDF)
