# Supplementary material for: Changes in cannabis policy and prevalence of recreational cannabis use among adolescents and young adults in Europe—An interrupted time-series analysis
Source: PLoS One. 2022 Jan 12;17(1):e0261885. doi: 10.1371/journal.pone.0261885 (PMC8754285; doi:10.1371/journal.pone.0261885)
Supplement: S1 File — (DOCX) [file pone.0261885.s001.docx]

## **S1 File. References describing methods for individual surveys.**

European Monitoring Centre for Drugs and Drug Addiction. (2019c). Statistical Bulletin 2019 — prevalence of drug use > Methodology.

Scientific institute of public health. Belgian health interview survey. Brussels (Belgium): sciensano. [visited 01-07-2020].

- Data on Belgian population’s self-reported cannabis use aged between 15 to 64 years, measured in subgroups of “ever used during lifetime”, “recent use” equal last 12 months, “current use” equal last 30 days, and presented in pools of ten years (15-24, 25-34, 35-44, 45-54, 55-64 year-olds).

Drugs and Drug Addictions Secretariat of the Council of the Government for Drug Policy Coordination Office. Annual Report: Czech Republic Drug Report. National Monitoring Centre for Drugs and Addiction. [visited 01-07-2020].

- Data on population self-reported cannabis use between 15 to 64 years of age, measured in subgroups of “ever used during lifetime”, “recent use” equal last 12 months, “current use” equal last 30 days, and presented in total or younger group (15-34 year-olds).

Herbst K, Kraus L, Scherer K. **Repräsentativerhebung zum Gebrauch psychoaktiver Substanzen bei Erwachsenen in Deutschland. Schriftliche Erhebung 1995 [Representative survey on use of psychoactive substances in adults in Germany. Written survey 1995].** Bundesministerium für Gesundheit. 1996.

- Data on German population’s self-reported cannabis use ages between 18 to 59 years, measured as “use last 12 months” and presented in total prevalence.

Kraus L, Bauernfeind R. Population Survey on the Consumption of Psychoactive Substances in the German Adult Population 1997. SUCHT. 1998; vol 44(1). ISSN 0939-5911.

- Data on German population’s self-reported cannabis use ages between 18 to 59 years, measured in subgroups of “ever used during lifetime”, “used last 12 months”, “used last 30 days”, and presented in total prevalence for East Germany respectively West Germany, as well as age groups of 18-20, 21-24, 25-39, 30-39, 40-49, and 50-59 year-olds.

Kraus L, Bauernfeind R. Population Survey on the Consumption of Psychoactive Substances in the German Adult Population 2000. SUCHT. 2001; vol 47(1). ISSN 0939-5911.

- Data on German population’s self-reported cannabis use ages between 18 to 59 years, measured in subgroups of “ever used during lifetime”, “used last 12 months”, “used last 30 days”, and presented in total prevalence for East Germany respectively West Germany, as well as age groups of 18-20, 21-24, 25-39, 30-39, 40-49, and 50-59 year-olds.

Kraus L, Augustin R, Orth B. Illicit drugs use, age of onset and trends. Results of the 2003 Epidemiological Survey of Substance Abuse. SUCHT. 2005; vol 51(1): pp19-28. DOI 10.1463/2004.07.01.

- Data on German population’s self-reported cannabis use ages between 18 to 59 years, measured in subgroups of “ever used during lifetime”, “used last 12 months”, “used last 30 days”, and presented in total prevalence, as well as age groups of 18-20, 21-24, 25-39, 30-39, 40-49, and 50-59 year-olds.

Pabst A, Piontek D, Kraus L, Muller S. Substance Use and Substance Use Disorders. Results of the 2009 Epidemiological Survey of Substance Abuse. SUCHT. 2010; vol 56(5): pp327-36.

- Data on German population’s self-reported cannabis use ages between 18 to 59 years, measured in “used last 12 months” and presented in total prevalence, as well as age groups of 18-20, 21-24, 25-39, 30-39, 40-49, and 50-59 year-olds.

Pabst A, Kraus L, Gomes E, Piontek D. Substanzkonsum und substanzbezogene Störungen in Deutschland im Jahr 2012 [Substance use and substance-related disorders in Germany 2012]. SUCHT. 2013; vol 59(6): pp321-21.

- Data on German population’s self-reported cannabis use ages between 18 to 59 years, measured in “used last 12 months” and presented in total prevalence, as well as age groups of 18-20, 21-24, 25-39, 30-39, 40-49, and 50-59 year-olds.

Gomez E, Atzendorf J, Kraus L, Piontek D. Substanzkonsum in der Allgemeinbevölkerung in Deutschland - Ergebnisse des Epidemiologischen Suchtsurveys 2015 [Substance use among general population in Germany – results of epidemiological addiction survey 2015]. SUCHT. 2016; vol 62(5): pp271-81. DOI 10.1024/0939-5911/a000445.

- Data on German population’s self-reported cannabis use ages between 18 to 64 years, measured in “used last 12 months” and presented in total prevalence.

MINISTERO DEL LAVORO E DELLE POLITICHE SOCIALI. RELAZIONE ANNUALE AL PARLAMENTO SULLO STATO DELLE TOSSICODIPENDENZE IN ITALIA 2002 [Annual report to parliament on the state of drug addiction in Italy 2002]. Ministry of labour and social policies. 2002.

- Data on Italian population’s self-reported cannabis use ages between 15 to 44 years, measured in “ever used during lifetime”, “used last 12 months”, used last 30 days”, and presented in total prevalence as well as age groups of 15-24, 25-34, 35-44 year-olds.

MINISTERO DEL LAVORO E DELLE POLITICHE SOCIALI. RELAZIONE ANNUALE AL PARLAMENTO SULLO STATO DELLE TOSSICODIPENDENZE IN ITALIA 2003 [Annual report to parliament on the state of drug addiction in Italy 2003]. Ministry of labour and social policies. 2003.

- Data on Italian population’s self-reported cannabis use ages between 15 to 44 years, measured in “ever used during lifetime”, “used last 12 months”, “used last 30 days”, and presented in total prevalence as well as age groups of 15-24, 25-34, 35-44 year-olds.

Istitutio Di Fisiologia Clinica – Sezione di epidemiologica e ricerca sui servizi sanitari. IPSAD – Italian Population Survey on Alcohol and other Drugs. Pisa (Italy): [upd 2019].

- Data on Italian population’s self-reported cannabis use ages between 15 to 64 years, measured in “ever used during lifetime”, “used last 12 months”, “used last 30 days”, and presented in total prevalence as well as age groups of 15-24, 25-34, 35-44, 45-54, 55-64 year-olds.

Abraham MD, Kaal HL, Cohen PDA. LICIT AND ILLICIT DRUG USE IN AMSTERDAM, 1987 TO 2001. CEDRO. 2003. ISBN 90 5330 3790.

- Data on Netherlands population’s self-reported cannabis use ages from 12 years old, measured in “lifetime prevalence”, “used last 12 months”, “used last month”, and presented in total prevalence as well as age groups of 12-15, 16-19, 20-24, 25-29, 30-34, 35-39, 40-49, 50-59, 60-69, 70 and older.

Laar MV, Cruts G, Gageldonk AV, Croes E, Ooyen-Houben MV, Meijer R et al. REPORT ON THE DRUG SITUATION 2006. Reitox national focal point. 2007. ISBN 978-90-5253-576-0.

- Data on Netherlands population’s self-reported cannabis use ages from 12 years old, measured in “lifetime prevalence”, “used last 12 months”, and presented in total prevalence as well as age groups of 15-24, 25-44, 45-64 year-olds.

Rooij AJV, Schoenmakers TM, Mheen DVD. Nationaal Prevalentie Onderzoek Middelengebruik 2009: De kerncijfers [National prevalence survey on substance use 2009: key figures]. Rotterdam IVO. 2011.

- Data on Netherlands population’s self-reported cannabis use ages between 15 to 64 years, measured in “lifetime prevalence”, “used last 12 months”, “used last month”, and presented in total prevalence.

Rijksinstituut voor Volksgezondheid en Milieu - Ministerie van Volksgezondheid, Welzijn en Sport. Drugs onder volwassenen [Drugs among adults]. RIVM. [upd 11-03-2020 08:14].

- Data on Netherlands population’s self-reported cannabis use ages from 18 years old, measured in “lifetime prevalence” and presented in total prevalence, as well as in age groups of 18-34, 35-49, 50-64, and 65 or older.

Norwegian Institute of Public Health. Drug Situation in Norway 2010. SIRUS. 2011. ISSN: 1503-1861.

- Data on Norwegian population’s self-reported cannabis use between 15 to 64 years, measured in “lifetime prevalence”, “used last 12 months”, “used last month”, and presented in total prevalence as well as in age groups of 15-24, 25-34, 35-44, 45-54, 55-64 years-olds.

Norwegian Institute of Public Health. Drug Situation in Norway 2011. SIRUS. 2011. ISSN: 1503-1861.

- Data on Norwegian population’s self-reported cannabis use between 15 to 64 years, measured in “lifetime prevalence”, “used last 12 months”, “used last month”, and presented in total prevalence as well as in age groups of 15-24, 25-34, 35-44, 45-54, 55-64 years-olds.

Norwegian Institute of Public Health. Drug Situation in Norway 2014. SIRUS. 2015. ISBN: 978-82-7171-422-2.

- Data on Norwegian population’s self-reported cannabis use between 15 to 64 years, measured in “lifetime prevalence”, “used last 12 months”, “used last month”, and presented in total prevalence as well as in age groups of 15-24, 25-34, 35-44, 45-54, 55-64 years-olds.

Norwegian Institute of Public Health. Alcohol and other psychoactive substances in Norway. Norwegian Institute of Public Health. [upd 24-05-2019].

- Data on Norwegian population’s self-reported cannabis use between 16 to 64 years, measured in “lifetime prevalence”, “used last 12 months”, “used last month”, and presented in total prevalence as well as in age groups of 16-24, 25-34, 35-44, 45-54, 55-64 years-olds.

Centro de Estudos de Sociologia da Universidade Nova de Lisboa. Ill INQUERITO NACIONAL ao Consumo de Substancias Psicoativas na Populacao Geral – Portugal 2012 [National survey on use of psychoactive substances in general population – Portugal 2012]. SICAD. 2014. ISBN 978-972-9345-87-6.

- Data on Portugal population’s self-reported cannabis use between 15 to 64 years, measured in “lifetime prevalence”; “used last year”, “used last month”, and presented in total prevalence as well as in the age groups of 15-34 and 35-64 years.

Centro de Estudos de Sociologia da Universidade Nova de Lisboa. Ill INQUERITO NACIONAL ao Consumo de Substancias Psicoativas na Populacao Geral – Portugal 2016/17 [National survey on use of psychoactive substances in general population – Portugal 2016/17]. SICAD. 2018. ISBN: 978-989-99574-9-7.

- Data on Portugal population’s self-reported cannabis use between 15 to 64 years, measured in “lifetime prevalence”; “used last year”, “used last month”, and presented in total prevalence as well as in the age groups of 15-34 and 35-64 years.

INFODROGY. STAV DROGOVEJ PROBLEMATIKY NA SLOVENSKU [State of drug problem in Slovakia]. Ministerstvo Zdravotnica SR. 2019; [visited 01-07-2020].

- Data on Slovenian population’s self-reported cannabis use between 15 to 95 years, measured in “lifetime prevalence”; “used last year”, “used last month”, and presented in total prevalence as well as age groups of 15-24, 15-64, 65-95 years.

Megias E. Los valores de la sociedad espanola y su relacion con las drogas [Values in Spanish society and its relationship with drugs]. Fundacion la Caixa. 2000.

- Data on Spanish population’s self-reported cannabis use between 15 to 65 years, measured in “lifetime prevalence”, “used last 12 months”, “habitual use” (not specified frequency), and presented in total prevalence as well as age groups of 15-19 and 20-29 year-olds.

Ministerio del Interior. “Spain” – Drug situation 2002. Reitox national focal point. 2002.

- Data on Spanish population’s self-reported cannabis use between 15 to 65 years, measured in “lifetime prevalence”, “used last 12 months”, “last month use”, and presented in total prevalence as well as age groups of 15-19 and 20-29 year-olds.

Delegación del Gobierno para el Plan Nacional sobre Drogas. ENCUESTA ESTATAL SOBRE USO DE DROGAS EN ENSEÑANZAS SECUNDARIAS (ESTUDES), 1994-2006 [State survey on the use of drugs in secondary schools, 1994-2006]. Gobierno para el Plan Nacional sobre Drogas. 2006. Data on Spanish population’s self-reported cannabis use between 14 to 18 years, measured in “lifetime prevalence”, “used last 12 months”, “last month use”, and presented in total prevalence.

EMCDDA. Resumen – Informe ESPAD 2007 [Summary – ESPAD report 2007]. EMCDDA. 2009.

- Data on Spanish children’s self-reported cannabis use between 15 and 16 years of age, measured in “lifetime prevalence”, “used last 12 months”, “used last month”, and presented in total prevalence.

Ministerio de Sandidad, Politica Social e Igualdad. SPAIN – New Development, Trends and in-depth information on selected issues. Reitox. 2010.

- Data on Spanish population’s self-reported cannabis use between 15 to 64 years, measured in “lifetime prevalence”, “used last 12 months”, “last month use”, and presented in total prevalence as well as age groups of 15-24, 25-34, 35-44, 45-54, 55-64 year-olds.

Ministerio de Sandidad. Plan Nacional sobre Drogas [National plan on drugs]. Gobierno de Espana. [visited 01-07-2020].

- Data on Spanish population’s self-reported cannabis use between 15 to 64 years, measured in “lifetime prevalence”, “used last 12 months”, “last month use”, “daily use”, and presented in total prevalence as well as age groups of 15-24, 25-34, 35-44, 45-54, 55-64 year-olds.

Folkhälsomyndigheten. Cannabisanvändning (självrapporterat) efter ålder, kön och år [Cannabis use (self-reported) by age, sex and year]. Folkhälsomyndigheten. [visited 01-07-2020].

- Data on Swedish population’s self-reported cannabis use between 16 to 84 years, measured in “lifetime prevalence”, “used last 12 months”, “last month use”, and presented in total prevalence as well as age groups of 16-29, 30-44, 45-64, 65-84 year-olds.

UK government. Drug missuse statistics. Home Office. [ upd 19-09-2019].

- Data on United Kingdom’s population’s self-reported cannabis use between 16 to 59 years, measured in “lifetime prevalence”, “used last 12 months”, “last month use”, and presented in total prevalence as well as the age group of 16-24 year-olds.
