# Supplementary material for: Changes in cannabis policy and prevalence of recreational cannabis use among adolescents and young adults in Europe—An interrupted time-series analysis
Source: PLoS One. 2022 Jan 12;17(1):e0261885. doi: 10.1371/journal.pone.0261885 (PMC8754285; doi:10.1371/journal.pone.0261885)
Supplement: S2 File — (DOCX) [file pone.0261885.s002.docx]

## **S2 File. List of implemented changes in cannabis legalisation changes during the study period, per studied country and calendar year.**

- Belgium
  - 2003: Personal possession was differentiated from possession of other controlled substances. In absence of aggravating circumstances, possession of cannabis for personal use is punishable with a fine.
- Czech Republic
  - 2009: A “criminal code” is implemented, which states that possession of a quantity of cannabis “greater than small” carries a prison sentence up to one year (instead of a fine before the implementation).
  - 2013: The supreme court interprets a “quantity greater than small” being equal to 10 grams of cannabis, put in relation of single dose usually being <1 gram.
- Italy
  - 2006: Differentiation of “soft” and “hard” drugs is removed, resulting in possession of cannabis becoming punishable as harshly as e.g., heroin.
  - 2014: Distinction is made between less dangerous, schedule II and IV, and more dangerous, schedule I and III, resulting in more lenient punishments for cannabis possession (first time offences usually only receive a warning).
- Norway
  - 2016: Implementation of a national rehabilitation program as alternative to a prison sentence, applicable under certain conditions.
- Portugal
  - 2001: Decriminalisation of cannabis use, possession as well as its acquisition for personal use.
- Spain
  - 2015: Supreme court dictates conditions under which cannabis clubs may operate, i.e. clubs open for new members or swift membership-changes are deemed as trafficking.
- United Kingdom
  - 2004: Cannabis is moved from being a class B drug, to the less grave class C. Possession of a class C drug is punishable with up to three month of imprisonment and/or a fine. On indictment, the penalty may be up to two years imprisonment and/or an unlimited fine.
  - 2009: Cannabis is reinserted as a class B drug. Possession of a class B drug is punishable with up to three months of imprisonment and/or a fine. On indictment, the penalty may be up to five years of imprisonment and/or an unlimited fine.
