# Supplementary material for: Changes in cannabis policy and prevalence of recreational cannabis use among adolescents and young adults in Europe—An interrupted time-series analysis
Source: PLoS One. 2022 Jan 12;17(1):e0261885. doi: 10.1371/journal.pone.0261885 (PMC8754285; doi:10.1371/journal.pone.0261885)
Supplement: S3 File — (PDF) [file pone.0261885.s003.pdf]

```
      name:  <unnamed>
       log:  /Users/nicors/OneDrive - KI.SE/Mac/Research/alexander/analysis_ca
> nnabis_8jun21.smcl
  log type:  smcl
 opened on:   8 Jun 2021, 13:03:18
```

```
1 . use http://www.stats4life.se/data/cannabis.dta, clear

2 . *bysort id (year): gen tag = cond(_n==_N, 1, 0)
3 .
4 . * Average trend among those countries (Germany, Netherlands, Slovakia, Swede
  > n) + (Belgium, Norway, Portugal, Spain)
5 .
6 . mixed sp year || country: year  if inlist(country, "Czechia", "Italy", "UK")
  > != 1,  reml cov(unstructured)  nolog
```

```
Mixed-effects REML regression                   Number of obs     =         67
Group variable: country                         Number of groups  =          8
                                                Obs per group:
                                                              min =          4
                                                              avg =        8.4
                                                              max =         15
                                                Wald chi2(1)      =       3.20
Log restricted-likelihood = -124.51436          Prob > chi2       =     0.0736
```

sp	Coefficient	Std. err.	z	P>|z|	[95% conf. interval]	
year	.0797562	.0445748	1.79	0.074	-.0076087	.1671212
_cons	-154.3555	88.81216	-1.74	0.082	-328.4242	19.7131

Random-effects parameters	Estimate	Std. err.	[95% conf. interval]	
**country:** Unstructured				
var(year)	.0107887	.0079683	.0025368	.0458826
var(_cons)	42523.75	31633.46	9895.14	182743.2
cov(year,_cons)	-21.41778	15.87602	-52.5342	9.698647
var(Residual)	1.329559	.2606739	.9053591	1.952516

```
LR test vs. linear model: chi2(3) = 123.05            Prob > chi2 = 0.0000

Note: LR test is conservative and provided only for reference.
```

```
 7 . lincom year, cformat(%3.2f)

 ( 1)  [sp]year = 0
```

sp	Coefficient	Std. err.	z	P>|z|	[95% conf.	interval]
(1)	**0.08**	**0.04**	**1.79**	**0.074**	**-0.01**	**0.17**

```
 8 .
 9 . * Figure 1
10 .
11 . levelsof country , local(lc)
   `"Belgium"' `"Czechia"' `"Germany"' `"Italy"' `"Netherlands"' `"Norway"' `"Por
   > tugal"' `"Slovakia"' `"Spain"' `"Sweden"' `"UK"'

12 .
13 . qui foreach c of local lc {

14 .
15 . graph combine `listplot'

16 . graph export "/Users/nicors/OneDrive - KI.SE/Mac/Research/alexander/figure1.
   > jpg", as(jpg) name("Graph") quality(100) replace
   file /Users/nicors/OneDrive - KI.SE/Mac/Research/alexander/figure1.jpg saved
       as JPG format

17 . graph close _all

18 .
19 . * Table 1 A) Implementation of more lenient legislation
20 .
21 . keep if inlist(country, "Czechia", "Italy", "UK")== 1
   (67 observations deleted)

22 .
```

```
23 . capture drop yfi yfi_plus

24 . gen yfi = year - 2013 if country == "Czechia" & inrange(year, 2010, 2017)
   (29 missing values generated)

25 . replace yfi = year - 2014 if  country == "Italy" & inrange(year, 2007, 2017)
   (4 real changes made)

26 . replace yfi = year - 2004 if  country == "UK" & inrange(year, 1994, 2008)
   (12 real changes made)

27 .
28 . gen yfi_plus = (yfi>0)*(yfi-0) if yfi != .
   (13 missing values generated)

29 .
30 . mixed sp yfi yfi_plus || country : yfi yfi_plus , reml  nolog

   Mixed-effects REML regression                   Number of obs     =         24
   Group variable: country                         Number of groups  =          3
                                                   Obs per group:
                                                                 min =          4
                                                                 avg =        8.0
                                                                 max =         12
                                                   Wald chi2(2)      =       0.19
   Log restricted-likelihood = -49.870326          Prob > chi2       =     0.9076
```

sp	Coefficient	Std. err.	z	P>|z|	[95% conf.	interval]
yfi	.0303394	.1489911	0.20	0.839	-.2616778	.3223567
yfi_plus	-.248681	.5654956	-0.44	0.660	-1.357032	.85967
_cons	9.426256	1.19018	7.92	0.000	7.093545	11.75897

Random-effects parameters	Estimate	Std. err.	[95% conf.	interval]
**country:** Independent				
var(yfi)	4.47e-10	1.09e-08	6.58e-31	3.04e+11
var(yfi_plus)	.501902	.7880028	.0231315	10.89014
var(_cons)	3.146698	3.670072	.3199469	30.94798
var(Residual)	2.773069	.9736205	1.393494	5.51844

LR test vs. linear model: chi2(3) = 5.71   Prob > chi2 = 0.1267

Note: LR test is conservative and provided only for reference.

```
31 .
32 . lincom yfi, cformat(%3.2f)

 ( 1)  [sp]yfi = 0
```

sp	Coefficient	Std. err.	z	P>|z|	[95% conf.	interval]
(1)	**0.03**	**0.15**	**0.20**	**0.839**	**-0.26**	**0.32**

```
33 . lincom yfi + yfi_plus, cformat(%3.2f)

 ( 1)  [sp]yfi + [sp]yfi_plus = 0
```

sp	Coefficient	Std. err.	z	P>|z|	[95% conf.	interval]
(1)	**-0.22**	**0.51**	**-0.43**	**0.667**	**-1.21**	**0.77**

```
34 . capture drop av_fit c_fit b*

35 . predictnl av_fit = predict() , ci(lo hi)
   (13 missing values generated)
   note: confidence intervals calculated using Z critical values.

36 . predict c_fit , fitted
   (13 missing values generated)

37 . predict b*, reffects relevel(country)

38 .
39 . * Country-specific BLUP based on the model
40 .
41 . levelsof country , local(lc)
   `"Czechia"' `"Italy"' `"UK"'
```

```
42 . foreach c of local lc {
  2.         qui su b1 if country == "`c'"
  3.         scalar blup_b1 = r(mean)
  4.         qui su b2 if country == "`c'"
  5.         scalar blup_b2 = r(mean)
  6.         noi di  _n "`c'"
  7.         display  "Trend before/after  = " %3.2f [(_b[yfi]+blup_b1)]*1 _c
> ///
>            "  "  %3.2f [(_b[yfi]+blup_b1) + (_b[yfi_plus]+blup_b2)]*1
  8. }

Czechia
Trend before/after  = 0.03  -0.25
Italy
Trend before/after  = 0.03  0.39
UK
Trend before/after  = 0.03  -0.79
43 .
44 . twoway  (scatter sp yfi  if country == "Czechia", mc(red%10) msize(small)) /
> //
>                (scatter sp yfi  if country == "Italy", mc(green%10)  msize(
> small)) ///
>                (scatter sp yfi  if country == "UK", mc(blue%10)  msize(smal
> l)) ///
>                (rarea lo hi yfi, fc(gs10%20) lc(gs10%20) sort ) ///
>                (line av_fit yfi, sort lc(black%80) lw(thick)) ///
>                (line c_fit yfi  if country == "Czechia", lc(red%30) sort) /
> //
>                (line c_fit yfi  if country == "Italy", lc(green%30) sort) /
> //
>                (line c_fit yfi  if country == "UK", lc(blue%30) sort) ///
>                , legend(label(1 "Czechia") label(2 "Italy") label(3 "UK") r
> egion(style(none))  order(1 2 3) ring(1) pos(4) col(1)) ///
>                ytitle("Self-reported cannabis use (%)") ///
>                ylabel(4(2)16, angle(horiz)) xline(0, lp(dash) lc(grey%50))
> xlabel(-10(1)4) ///
>                xtitle("Years from intervention") ///
>                plotregion(style(none)) ///
>                title("A) Implementation of more lenient legislation") ///
>                name(figure1A, replace)
(note:  named style grey not found in class color, default attributes used)
```

```
.
. graph export figure1A.pdf, as(pdf) replace
file /Users/nicors/OneDrive - KI.SE/Mac/Research/alexander/figure1A.pdf
    saved as PDF format

.
. * Table 1. B)   Implementation of stricter legislation
.
. capture drop yfi yfi_plus

. gen yfi = year - 2009 if country == "Czechia" & inrange(year, 2004, 2012)
(31 missing values generated)

. replace yfi = year - 2006 if  country == "Italy" & inrange(year, 2001, 2013)
(5 real changes made)

. replace yfi = year - 2009 if  country == "UK" & inrange(year, 2005, 2017)
(11 real changes made)

.
. gen yfi_plus = (yfi>0)*(yfi-0) if yfi != .
(15 missing values generated)

.
. mixed sp yfi yfi_plus || country :  yfi yfi_plus , reml  nolog

Mixed-effects REML regression                   Number of obs     =         22
Group variable: country                         Number of groups  =          3
                                                Obs per group:
                                                              min =          5
                                                              avg =        7.3
                                                              max =         11
                                                Wald chi2(2)      =       3.87
Log restricted-likelihood = -47.145137          Prob > chi2       =     0.1446
```

sp	Coefficient	Std. err.	z	P>|z|	[95% conf.	interval]
yfi	.0078687	.3137429	0.03	0.980	-.6070561	.6227934
yfi_plus	-.4459111	.4594528	-0.97	0.332	-1.346422	.4545998
_cons	9.049132	.8752353	10.34	0.000	7.333703	10.76456

Random-effects parameters	Estimate	Std. err.	[95% conf.	interval]
**country:** Independent				
var(yfi)	**.0201629**	**.0927441**	**2.45e-06**	**165.9063**
var(yfi_plus)	**1.58e-12**	**8.55e-09**	**0**	**.**
var(_cons)	**.4785073**	**1.088806**	**.0055341**	**41.37443**
var(Residual)	**4.111024**	**1.482871**	**2.027293**	**8.336496**

LR test vs. linear model: chi2(**3**) = **0.42** Prob > chi2 = **0.9352**

Note: LR test is conservative and provided only for reference.

```
58 .
59 . lincom yfi, cformat(%3.2f)
```

(1) **[sp]yfi = 0**

sp	Coefficient	Std. err.	z	P>|z|	[95% conf.	interval]
(1)	**0.01**	**0.31**	**0.03**	**0.980**	**-0.61**	**0.62**

```
60 . lincom yfi + yfi_plus, cformat(%3.2f)
```

(1) **[sp]yfi + [sp]yfi_plus = 0**

sp	Coefficient	Std. err.	z	P>|z|	[95% conf.	interval]
(1)	**-0.44**	**0.24**	**-1.83**	**0.067**	**-0.91**	**0.03**

```
61 . capture drop av_fit c_fit b*
```

```
62 . capture drop lo hi

63 . predictnl av_fit = predict() , ci(lo hi)
   (15 missing values generated)
   note: confidence intervals calculated using Z critical values.

64 . predict c_fit , fitted
   (15 missing values generated)

65 . predict b*, reffects relevel(country)

66 .
67 . * Country-specific BLUP based on the model
68 .
69 . levelsof country , local(lc)
   `"Czechia"' `"Italy"' `"UK"'

70 . foreach c of local lc {
     2.         qui su b1 if country == "`c'"
     3.         scalar blup_b1 = r(mean)
     4.         qui su b2 if country == "`c'"
     5.         scalar blup_b2 = r(mean)
     6.         noi di  _n "`c'"
     7.         display  "Trend before/after  = " %3.2f [(_b[yfi]+blup_b1)]*1 _c
   > ///
   >            "  "  %3.2f [(_b[yfi]+blup_b1) + (_b[yfi_plus]+blup_b2)]*1
     8. }

   Czechia
   Trend before/after  = -0.04  -0.49
   Italy
   Trend before/after  = 0.08  -0.36
   UK
   Trend before/after  = -0.02  -0.46
71 .
72 . twoway  (scatter sp yfi  if country == "Czechia", mc(red%10) msize(small)) /
   > //
   >                 (scatter sp yfi  if country == "Italy", mc(green%10)  msize(
   > small)) ///
   >                 (scatter sp yfi  if country == "UK", mc(blue%10)  msize(smal
   > l)) ///
   >                 (rarea lo hi yfi, fc(gs10%20) lc(gs10%20) sort ) ///
   >                 (line av_fit yfi, sort lw(thick) lc(black%80)) ///
   >                 (line c_fit yfi  if country == "Czechia", lc(red%30) sort) /
   > //
   >                 (line c_fit yfi  if country == "Italy", lc(green%30) sort) /
   > //
   >                 (line c_fit yfi  if country == "UK", lc(blue%30) sort) ///
   >                 , legend(label(1 "Czechia") label(2 "Italy") ///
```

```
>                 label(3 "UK") region(style(none))  order(1 2 3) ring(1) pos(
> 4) col(1)) ///
>                 ytitle("Self-reported cannabis use (%)") ///
>                 ylabel(4(2)16, angle(horiz)) xline(0, lp(dash) lc(grey%50))
> xlabel(-5(1)8) ///
>                 xtitle("Years from intervention") ///
>                 title("B) Implementation of more stricter legislation") ///
>                 plotregion(style(none)) ///
>                 name(figure1B, replace)
(note:  named style grey not found in class color, default attributes used)

73 .
74 . graph export figure1B.pdf, as(pdf) replace
file /Users/nicors/OneDrive - KI.SE/Mac/Research/alexander/figure1B.pdf
    saved as PDF format

75 .
76 . log close
      name:  <unnamed>
       log:  /Users/nicors/OneDrive - KI.SE/Mac/Research/alexander/analysis_ca
> nnabis_8jun21.smcl
  log type:  smcl
 closed on:   8 Jun 2021, 13:04:13
-------------------------------------------------------------------------------
```
